# Global patterns of prescription pain medication usage in disorders of gut–brain interactions

**DOI:** 10.1111/nmo.14457

**Published:** 2022-09-16

**Authors:** Yuying Luo, Suzi A. Camey, Shrikant I. Bangdiwala, Olafur S. Palsson, Ami D. Sperber, Laurie A. Keefer

**Affiliations:** ^1^ The Dr. Henry D. Janowitz Division of Gastroenterology Icahn School of Medicine at The Mount Sinai Hospital New York City New York USA; ^2^ Hospital de Clínicas de Porto Alegre Universidade Federal do Rio Grande do Sul Porto Alegre Brazil; ^3^ Department of Health Research Methods, Evidence and Impact McMaster University Hamilton Ontario Canada; ^4^ Population Health Research Institute, McMaster University Hamilton Ontario Canada; ^5^ Center for Functional GI & Motility Disorders University of North Carolina Chapel Hill North Carolina USA; ^6^ Faculty of Health Sciences Ben‐Gurion University of the Negev Beer‐Sheva Israel

**Keywords:** disorders of gut‐brain interaction, pain, prescription pain medications

## Abstract

**Background:**

Forty percent of individuals globally meet Rome IV criteria for a disorder of gut–brain interaction (DGBI). The global burden of pain across these disorders has not been characterized.

**Methods:**

Our study included 54,127 respondents from the 26 Internet survey countries. Prescription pain medication usage was selected as the proxy for pain. The associations between prescription pain medications and the environmental, sociodemographic, psychosocial, and DGBI diagnosis variables were investigated using the multivariate generalized robust Poisson regression model.

**Key Results:**

Respondents with DGBI used prescription pain medications at higher rates than those without a DGBI diagnosis with pooled prevalence rate of 14.8% (95% confidence interval [CI], 14.4–15.3%), varying by country from 6.8% to 25.7%. The pooled prevalence ratio of prescription pain medication usage in respondents with and without DGBI was 2.2 (95% CI: 2.1–2.4). Factors associated with higher prevalence of pain medication usage among respondents with a DGBI diagnosis included living in a small community, increased anxiety, depression or somatization, increased stress concern or embarrassment about bowel functioning and having more than one anatomic DGBI diagnosis.

**Conclusion:**

14.8% of patients globally with at least one diagnosis of DGBI were on prescription pain medications with wide geographic variation, about twice as many as their counterparts without a diagnosis of DGBI. Environmental, sociodemographic, and individual factors may influence clinicians to consider personalized, multimodal approaches to address pain in patients with DGBI.

## Graphical abstract

14.8% of respondents globally who have **≥**1 DGBI diagnosis were on prescription pain medications, ~2× as high as respondents without a DBGI diagnosis. Wide geographic variation, but pain medication prescription patterns in DGBI are influenced by overall national prescribing behaviors.

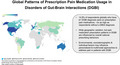

## 1 INTRODUCTION

Pain throughout the gastrointestinal (GI) tract affects a significant proportion of the population and is a central feature of many functional gastrointestinal disorders (FGID), now known as disorders of gut–brain interaction (DGBI).^1^ Chronic GI pain results in significant global healthcare costs and impaired health‐related quality of life (QoL). A variety of mechanisms are posited to cause visceral pain including disordered GI motility and sensation stemming from peripheral (e.g., post‐infection inflammation, luminal irritants, and immunogenic responses to environmental exposures) and centrally mediated (e.g., stress and anxiety) factors.^2^ The treatment of pain in the context of DGBI is especially challenging given its multifactorial nature.

Until recently, the global prevalence and distribution of DGBI were unknown. The Rome Foundation Global Epidemiology Study (RFGES), a seminal epidemiologic study conducted simultaneously in 33 countries that assessed the worldwide prevalence and burden of DGBIs, found that 40.3% of Internet surveyed individuals met Rome IV criteria for a DGBI.^3^ This has pronounced implications when considering the economic burden on healthcare systems and impact on quality of life.

Although “pain” is explicitly included in the Rome IV criteria for the diagnosis of many DGBIs, the global burden of pain across these disorders has not been characterized. We sought to characterize the prevalence of prescription pain medication use in patients with at least one DGBI diagnosis across the surveyed countries. We also aimed to assess trends in pain medication usage in patients with DGBI across regions and different anatomic groups of DGBIs. Finally, we evaluated clinical and sociodemographic factors associated with prescription pain medication usage in patients with DGBI.

## 2 MATERIALS AND METHODS

The RFGES survey methods were previously described in detail.^3^ Briefly, data were collected by Internet survey only in 24 countries, by personal interview in seven countries and by both methods in two countries (China and Turkey). The predefined demographic parameters for all countries were 50% female and 50% male individuals, and 40% for 18–39 years, 40% for 40–64 years, and 20% for 65+ years, including over 76,000 individuals globally.

Our study included 54,127 respondents from the 26 Internet survey countries, 21,716 who met the criteria for at least one DGBI diagnosis. We excluded missing values from the analysis which entailed 695 (3.2%) respondents. Prescription pain medication usage was assessed with the question: “Are you currently taking a prescription medication for pain?” This question encompasses the spectrum of prescription medications for pain which included both opioid and non‐opioid analgesics (e.g., NSAIDs, neuromodulators, medications prescribed by traditional healers). Prescription pain medication was selected as the primary dependent variable as this was a rigorous and objective dichotomous measure of pain which was correlated with overall body pain in the past week in respondents with at least one DGBI diagnosis; the mean pain score was 6.44 (6.37–6.52) for prescription pain medication users versus 4.43 (4.40–4.47) [*p* < 0.001] for those not taking pain medication. For evaluation of health‐related QoL, the Global Physical Health, and Global Mental Health summary scores were derived from the PROMIS Global‐10 questionnaire, with higher scores indicating higher QoL.^4^

Categorical and numerical variables were summarized by descriptive statistics and reported along with 95% confidence intervals. Prevalence ratios for each surveyed country were determined by calculating the ratio of prescription medications usage in respondents with and without a diagnosis of DGBI. For respondents with at least one DGBI diagnosis, the associations (and adjusted prevalence ratios) between prescription pain medications and the environmental, sociodemographic, psychosocial, and DGBI diagnosis variables were investigated using the multivariate generalized robust Poisson regression model. The effects of potential confounders were analyzed following the conceptual hierarchical framework^5^; in each step, variables with *p* > 0.20 were dropped from the next model except for sex given its clinical relevance (Figure 2).

All statistical analyses were conducted using SPSS (version 18) and R (version 4.1).

## 3 RESULTS

### 3.1 Pain prescription patterns by country

The prevalence rates and prevalence ratios of prescription pain medications use by patients with or without at least one DGBI diagnosis are shown in Table 1.

**TABLE 1:** Prevalence rates (% and 95% CI) of prescription pain medication use of individuals with ≥1 disorder of gut–brain interaction (DGBI) diagnosis and prevalence ratios compared to individuals without a DGBI diagnosis

Country	*n*	Prevalence	Prevalence ratios
Internet			
Argentina	904	8.3 (6.6–10.3)	2.0 (1.4–2.9)
Australia	765	22.2 (19.3–25.3)	1.9 (1.5–2.3)
Belgium	719	16.1 (13.5–19.0)	2.3 (1.8–2.9)
Brazil	874	13.6 (11.4–16.1)	2.3 (1.8–3.1)
Canada	837	16.7 (14.3–19.4)	1.8 (1.4–2.3)
China	1002	13.4 (11.3–15.6)	3.5 (2.7–4.6)
Colombia	853	10.4 (8.5–12.7)	2.1 (1.5–2.9)
Egypt	962	17.3 (14.9–19.8)	2.7 (2.0–3.5)
France	953	22.9 (20.2–25.7)	2.6 (2.1–3.2)
Germany	738	17.9 (15.2–20.9)	1.6 (1.3–2.0)
Israel	732	6.8 (5.1–8.9)	2.5 (1.6–3.8)
Italy	912	15.0 (12.8–17.5)	2.0 (1.6–2.6)
Japan	987	8.9 (7.2–10.9)	3.2 (2.3–4.6)
Mexico	804	19.3 (16.6–22.2)	2.2 (1.8–2.8)
Netherlands	614	13.4 (10.8–16.3)	2.3 (1.7–3.0)
Poland	947	14.4 (12.2–16.8)	1.9 (1.5–2.5)
Romania	821	9.4 (7.5–11.6)	3.6 (2.4–5.4)
Russia	892	11.4 (9.4–13.7)	3.0 (2.1–4.3)
Singapore	636	6.8 (4.9–9.0)	3.2 (2.0–5.0)
South Africa	913	15.3 (13.1–17.8)	2.6 (2.0–3.5)
South Korea	795	8.1 (6.3–10.2)	2.9 (1.9–4.4)
Spain	906	17.0 (14.6–19.6)	2.2 (1.7–2.8)
Sweden	812	16.1 (13.7–18.9)	1.3 (1.0–1.6)
Turkey	798	14.8 (12.4–17.4)	2.3 (1.7–3.0)
United States	807	24.5 (21.6–27.7)	2.2 (1.8–2.6)
United Kingdom	743	25.7 (22.6–29.0)	2.3 (1.9–2.8)

The prevalence of pain medication usage in patients with at least one DGBI diagnosis ranged widely for survey countries with pooled prevalence rate of 14.8% (14.4%–15.3%). Singapore at 6.8% (4.9%–9.0%) and Israel at 6.8% (5.1%–8.9%) had the lowest rates of pain medication use whereas United States and the United Kingdom had the highest rates at 24.5% (21.6%–27.7%) and 25.7% (22.6%–29%), respectively.

We also calculated the prevalence ratios of prescription pain medication use in respondents with at least one DGBI diagnosis compared with those without a single DGBI diagnosis to better understand overall pain prescription patterns by country. The pooled prevalence ratio was 2.2 (2.1–2.4), varying from 1.3 for Sweden (1.0–1.6) to 3.6 for Romania (2.4–5.4). Despite differences in prevalence rates of prescription medication usage in respondents with DGBI between the high and low prevalence countries as noted above, the corresponding prevalence ratios were more similar than different in these countries; the prevalence ratios were 3.2 (2.0–5.0) for Singapore, 2.5 (1.6–3.8) for Israel, 2.2 (1.8–2.6) for the United States, and 2.3 (1.9–2.8) for the United Kingdom.

### 3.2 Prevalence of pain medication usage by specific DGBI anatomic regions and diagnoses

Based on the accepted Rome IV categorization, the GI tract was divided into four anatomic regions (esophageal, gastroduodenal, bowel, and anorectal). Two DGBI categories, centrally mediated abdominal pain and biliary pain, were not included in the analyses due to the low number of diagnosed individuals meeting criteria for these diagnoses (below 0.1% of the population surveyed).

**FIGURE 1:**
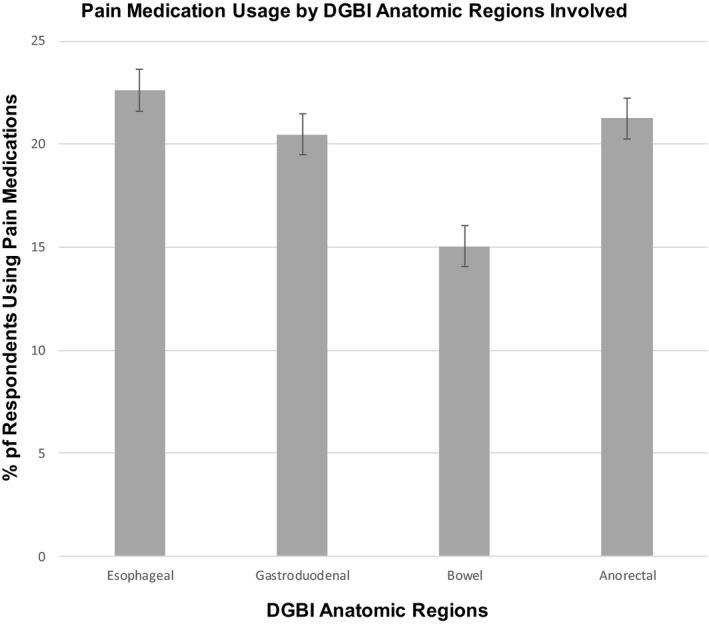
Pattern of pain medication usage across 26 internet survey countries by specific DGBI anatomic region involved. Pain medication usage was highest for esophageal‐related disorder of gut–brain interaction (DGBI) conditions at 22.6% (21.2%–24.1%), followed by anorectal‐related at 21.3% (20.0%–22.5%), gastroduodenal‐related at 20.5 (19.4%–21.5%), and lowest for bowel‐related DGBI conditions at 15.1% (14.5%–15.6%).

The pattern of pain medication usage by specific DGBI anatomic region involved is shown in Figure 1. Pain medication usage was highest for esophageal‐related DGBI conditions at 22.6% (21.2%–24.1%), followed by anorectal‐related at 21.3% (20.0%–22.5%), gastroduodenal‐related at 20.5 (19.4%–21.5%), and lowest for bowel‐related DGBI conditions at 15.1% (14.5%–15.6%).

**FIGURE 2:**
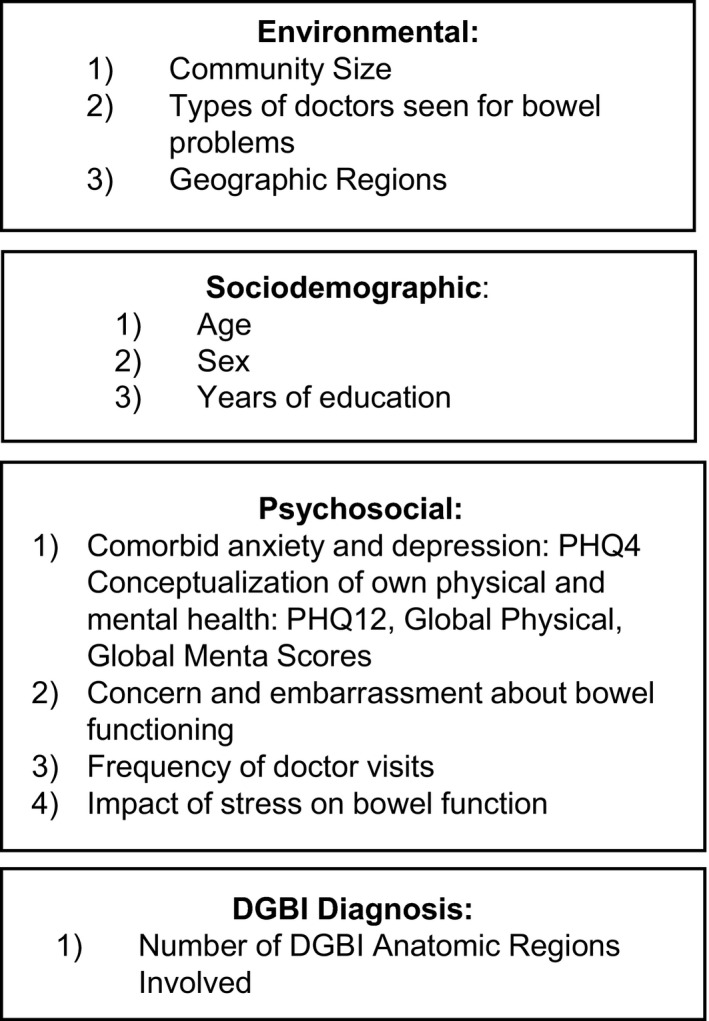
Conceptual hierarchical framework of environmental, sociodemographic, and individual factors which may affect pain prescription medication use in disorders of gut–brain interaction.

The prevalence rate for prescription medication usage for each Rome IV disorder is listed in Table 2. As expected, the prevalence of prescription medication usage for opioid‐induced constipation was 100%, confirming the internal validity of our selected dependent variable (“Are you currently taking a prescription medication for pain?”).

**TABLE 2:** Prevalence of prescription pain medication use (% and 95% CI) by Rome IV criteria disorders of gut‐brain interaction

Disorders of gut‐brain interaction	*n*	Prevalence
Functional heartburn	613	38.3 (34.5–42.3)
Functional chest pain	741	13.6 (11.2–16.3)
Reflux hypersensitivity	455	36.9 (32.5–41.5)
Globus sensation	408	10.8 (8.0–14.2)
Functional dysphagia	1712	26.9 (24.8–29.0)
Functional biliary pain	36	22.2 (10.1–39.2)
Functional dyspepsia	3910	22.3 (21.0–23.6)
Postprandial distress syndrome	3313	20.7 (19.3–22.1)
Epigastric pain syndrome	1306	34.5 (32.0–37.2)
Excessive belching, unspecified (may be supragastric or gastric)	525	25.5 (21.9–29.5)
Rumination	1511	16.6 (14.8–18.6)
Chronic nausea and vomiting	503	27.8 (24.0–32.0)
Cyclic vomiting diagnosed	624	34.6 (30.9–38.5)
Cannabinoid hyperemesis syndrome	28	67.9 (47.7–84.1)
Irritable bowel syndrome (IBS)	2195	26.2 (24.4–28.1)
IBS‐Constipation	740	27.4 (24.3–30.8)
IBS‐Diarrhea	657	26.0 (22.7–29.6)
IBS‐Mixed	748	29.4 (26.2–32.8)
IBS‐Unclassified	146	18.5 (12.6–25.8)
Functional constipation	6333	7.6 (7.0–8.3)
Opioid‐induced constipation	846	100.0 (99.6–100.0)
Functional bowel disorder, unspecified	4762	11.0 (10.1–11.9)
Functional diarrhea	2547	11.9 (10.6–13.2)
Functional bloating/distention	1785	12.4 (10.9–14.0)
Central Abdominal Pain Syndrome	9	33.3 (7.5–70.1)
Fecal incontinence	851	28.0 (25.0–31.1)
Levator ani syndrome	622	25.4 (22.0–29.0)
Proctalgia fugax	3013	20.4 (19.0–21.9)

Cannabinoid hyperemesis syndrome had the highest prevalence rate of prescription pain medications at 67.9% (47.7%–84.1%); however, this group only consisted of 28 respondents. Among functional esophageal disorders, the prevalence of prescription pain medications was highest for functional heartburn followed by reflux hypersensitivity. For gastroduodenal disorders, respondents with the epigastric pain syndrome subtype of functional dyspepsia had higher prevalence prescription pain medication usage compared with postprandial distress syndrome. The use of pain medications was higher in respondents with all subtypes of irritable bowel syndrome (IBS) compared with other functional bowel disorders (functional constipation, functional diarrhea, and functional bowel disorder, unspecified). For instance, the prevalence of prescription pain medication use for the IBS with predominant constipation subtype was 27.4% (24.3%–30.8%) compared with 7.6% (7.0%–8.3%) for functional constipation. The use of pain medications was similar across all functional anorectal disorders (fecal incontinence, levator ani syndrome, and proctalgia fugax).

### 3.3 Factors associated with prescription pain medication usage

The associations between environmental, sociodemographic, and psychosocial factors and the prevalence of pain medication usage among respondents with at least one DGBI diagnosis were investigated using a multivariate generalized robust Poisson regression model based on a conceptual hierarchical framework (Table 3).

**TABLE 3:** Adjusted prevalence ratios (and 95% CI) for factors associated with pain prescription usage

Model 1	
Community size ≤2500 inhabitants	1.16 (1.05–1.28)
What kind of doctors have you seen for bowel problems?	
General practitioner or family doctor	1.52 (1.40–1.62)
Gastroenterologist (a doctor who specializes in bowel problems)	1.67 (1.56–1.79)
Surgeon	1.51 (1.34–1.71)
Folk healer or traditional healer	1.17 (0.85–1.62)
Traditional Chinese Medicine doctor	1.13 (0.89–1.42)
Model 2	
Age	1.02 (1.01–1.02)
Sex	0.99 (0.93–1.06)
Years of education	0.98 (0.98–0.99)
Model 3	
PHQ‐4 score	1.03 (1.02–1.04)
PHQ‐12 score	1.04 (1.03–1.05)
Global physical health component score	0.85 (0.83–0.86)
Global mental health component score	1.07 (1.06–1.09)
Are you embarrassed to discuss your bowel functioning with others (family, friends)?	
Not at all	1
Somewhat	1.12 (1.05–1.20)
Very embarrassed	1.14 (1.03–1.25)
How often do you go to a doctor for your health?	
Once a month or more	1
A few times a year	0.66 (0.61–0.70)
Once a year	0.44 (0.38–0.50)
Less than once a year	0.28 (0.24–0.33)
Never	0.33 (0.24–0.47)
Does stress, pressure or tension affect your bowel functioning?	
Not at all	1
Somewhat	0.97 (0.90–1.05)
Greatly affects it	0.91 (0.83–1.00)
Model 4	
Number of DGBI Regions Involved	
1	1
2	1.17 (1.09–1.26)
3	1.25 (1.13–1.38)
4	1.56 (1.37–1.76)

Abbreviation: DGBI, disorder of gut–brain interaction.

Community size less than 2500, older age, seeing any type of practitioner for bowel‐related concerns (although seeing a general practitioner, gastroenterologist, and surgeon had the most impact), higher PHQ‐4 score for anxiety/depression, higher PHQ‐12 score for somatization, increased stress, concern, or embarrassment about bowel functioning and a higher frequency of doctor visits, were associated with higher prevalence of pain medication. Female sex was associated with a slightly lower prevalence of pain medication usage (prevalence ratio 0.99 for Internet countries).

A higher global physical health component score seemed protective against prescription pain medication usage for patients with DGBI, however, a higher global mental health component score was associated with a higher prevalence of pain medication usage.^4^

Increased prevalence of pain medication usage was seen with increasing numbers of overlapping DGBI anatomical regions. For Internet countries, compared with patients who had DGBI in only one anatomical region, the prevalence of prescription pain medications in respondents with DGBI (when accounting for all the variates above) was 17%, 25%, and 56% higher when the patient had DGBI involving 2, 3, or 4 anatomical regions.

## 4 DISCUSSION

This study is the first to rigorously characterize the global burden of pain in individuals with Rome IV DGBI diagnoses, with prescription pain medication serving as a proxy for pain. Our results highlight the substantial need for future research to address pain within the context of DGBI with both novel pharmacologic and non‐pharmacologic approaches.

Assessing pain in a large global study is inherently difficult given that pain exists on a heterogeneous spectrum. We selected prescription pain medication as a surrogate for pain as this was an objective, readily accessible, and dichotomous variable. This was an internally valid selection as prescription pain medication use was associated with a significantly higher mean global pain score compared with respondents not on prescription pain medication use (see Section 2) and the prevalence of patients with opioid‐induced constipation on prescription pain medication was 100%. Our study may actually underestimate the burden of pain as there are respondents with a diagnosis of DGBI who have pain but are not on prescription pain medications. The survey question did not specify the class of prescription pain medication and theoretically encompasses the spectrum of prescription medications for pain which included both opioid and non‐opioid analgesics (e.g., neuromodulators, medications prescribed by traditional healers). We attempted to clarify this by assessing prescription pain medication usage in patients with concomitant medication usage for depression or anxiety. Of patients with at least one DGBI diagnosis who reported weekly medication usage for depression or anxiety, 30.8% (29.1%–32.6%) and 28.7% (27.1%–30.4%) of patients, respectively, also reported taking prescription pain medication, compared with 12.5% (12.1%–13.0%) and 12.7% (12.2%–13.2%) of patients with at least one DGBI diagnosis who reported not taking weekly medications for depression and anxiety. However, medications for depression or anxiety are used for both psychiatric comorbidities and/or neuromodulation, highlighting the inherent biopsychosocial nature of DGBIs.

Respondents with at least one DGBI diagnosis were uniformly more likely than those respondents without a DGBI diagnosis to be on a prescription pain medication. Notably, the latter group could still have non‐GI diseases for which prescription pain medicine might be prescribed and does not represent a totally healthy control population. Despite this potential negative bias, the prevalence ratio of pain prescription medicine rates still demonstrated a higher prevalence in respondents with a DGBI diagnosis compared with those without. The lowest prevalence countries had fewer than 10% patients with DGBI on prescription pain medications in contrast to the highest prevalence countries which reported a quarter to a third of patients with a diagnosis DGBI on prescription pain medications. Asian countries (Korea, Japan, Singapore, China) and South American (Colombia, Argentina) had lower prevalence rates of pain medication usage (ranges 6.8%–13.4% and 8.3%–10.4%, respectively), among respondents with at least one DGBI diagnosis compared with North American countries (United States, Canada, and Mexico with range 16.7%–24.5%); prevalence rates within European countries varied more widely (range 6.8%–25.7%). However, we observed similar prevalence ratios for low and high prevalence countries, suggesting that national trends impact pain prescription patterns (i.e., in high prevalence countries, patients without a diagnosis of DGBI also have a higher prevalence of pain medication usage). The regional differences in prescription pain medication usage, particularly between lower prevalence countries in Asia and South America versus higher prevalence countries in North America may reflect differences in the social attitudes and cultural conceptualizations of DGBI and pain. There are also regional differences in treatment algorithms and approaches of providers in addressing pain (e.g., complementary alternative medications and non‐pharmaceutical modalities).

Increased prevalence of pain medication was seen with increased number of DGBI anatomic regions involved. A previous Rome Foundation Global study found that DGBI in multiple anatomic GI regions is associated with increased psychological comorbidity, healthcare utilization, and IBS severity.^6^ This is a focus of the “Overlap in DGBI” Rome Foundation working group whose goals are to advance current diagnostic and therapeutic approaches in patients with overlap among DGBI in the different regions in the GI tract, in addition with overlap with organic GI diseases and with non‐GI symptoms and syndromes.^7^

There were several notable patterns of prescription pain medication usage by specific DGBI diagnoses. Respondents with cannabinoid hyperemesis syndrome had a significantly higher rate of prescription pain medication usage compared other DGBIs; although the sample size was limited to 28 respondents, this pattern may reflect a higher prevalence of chronic pain in respondents who use cannabinoids as an adjunct to prescription pain medications. Among the four anatomic regions, the rates of prescription medication usage were highest for functional esophageal disorders, suggesting that these disorders may benefit from additional modalities for pain modulation.^8^ Patients with functional esophageal disorders often have overlap with proven gastroesophageal reflux disease or reflux hypersensitivity and initial treatments for patient's symptom burden may be directed at reflux instead of neuromodulation.^9^ The prevalence rates of prescription pain medication use were also higher for all IBS subtypes compared with other functional bowel disorders (e.g., functional constipation and diarrhea). This supports the inclusion of pain in the Rome IV criteria for IBS diagnoses and perhaps supports the separation of IBS‐C and functional constipation and IBS‐D and functional diarrhea as distinct clinical entities. We did not segregate specific DGBIs into “painful” or “non‐painful” groups as our data (Table 2) challenges the traditional conceptualization of some of these disorders. This dichotomy appears to hold true for some DGBIs, (e.g., respondents with the epigastric pain syndrome subtype of functional dyspepsia had higher prevalence prescription pain medication usage compared with postprandial distress); however, respondents with DGBI not commonly thought of as “painful” such as fecal incontinence (28.0%, 95% CI 25.0%–31.1%) and cyclical vomiting syndrome (34.6%, 95% CI 30.9%–38.5%) also reported considerable prescription pain medication usage.

Previous data found that the prevalence of FGIDs decreased with age in Internet survey countries^3^ (which may be related to age‐related decrease in abdominal pain perceptions^10^). We observed increased prevalence of prescription pain medication usage with age. Given the risk of polypharmacy in older adults, this is a population where we need to consider the utilization of non‐pharmacologic approaches. While there was a female predominance of DGBI in all regions of the GI tract as seen in previous studies,^3^, ^11^ interestingly, female sex in our study was associated with slightly lower prevalence of pain medication usage when adjusting for the above variable despite studies demonstrating that females have increased pain sensitivity and experience more severe clinical pain across a variety of conditions.^12^ This suggests that females may not be reporting their pain to their providers, or their pain may be perceived as less substantial by providers who refer them preferentially to nonpharmacologic approaches. Residing in a community with fewer than 2500 inhabitants was also associated with increased prevalence of prescription pain medication use; this may be secondary to more limited access to non‐pharmaceutical modalities of treating pain or other environmental factors which may exacerbate pain severity. More robust physical health was associated with lower prevalence of pain medication usage; however, the same was not seen with mental health, suggesting the complex interplay between biopsychological factors in treating pain within the context of DGBI.

These are important sociodemographic and clinical factors to consider for clinicians treating patients with DGBI and are part of the more holistic Rome Multidimensional Clinical Profile for early management of DGBI.^13^ These factors may also cue clinicians to consider non‐pharmacologic adjunct interventions earlier or up‐front such as cognitive behavioral therapies (CBT), hypnosis, and mindfulness meditation, which all have demonstrated varying degrees of efficacy in reducing pain and improving quality of life.^14^, ^15^, ^16^, ^17^, ^18^, ^19^ A recent pragmatic open‐label trial showed integrated multidisciplinary clinical care to be superior to gastroenterologist‐only care in relation to DGBI patient outcomes. Our data support that integrated care with a multimodal approach to address pain is key to potentially decreasing the pharmaceutical burden of chronic pain, especially in patients with risk factors as outlined above.^20^ However, access to integrated care is not universal and is vulnerable to disparities across regions and community sizes.

### 4.1 Limitations

There are limitations to our study, First, the lack of specificity under the umbrella category of “prescription pain medication” includes opioids and non‐opioid analgesics. Second, there were no questions regarding duration of prescription pain medication use or information linking pain medication usage for symptoms specifically attributable to a DGBI diagnosis. However, if participants screened positive for a checklist of organic diagnoses and surgeries that might account for other GI symptoms, they were disqualified from the DGBI group and included in the comparison group without DGBI. Our observations of a clear increased prevalence ratio of pain medication usage in patients with at least one DGBI diagnosis compared with those without across all surveyed countries suggests that DGBI was at least a contributing factor to overall pain burden on the individual‐level. It is currently unclear how a diagnosis of DGBI augments or modulates non‐GI pain and symptoms and this is a needed area of future research.^7^ Third, our study may also underestimate the prevalence of pain in respondents with DGBI as we only assessed prescription pain medication use. In one large cross‐sectional study in the United States, 81% of individuals experienced abdominal pain within the past week; however, two in five individuals did not seek care for their symptoms and many of them might have undiagnosed, treatable disorders.^21^

### 4.2 Future directions

Our results highlight the need for innovative pharmacologic and non‐pharmacologic approaches to address pain in patients with DGBI. In a recent analysis of US gastroenterologist prescribing patterns, 10% of outpatient GI visits were associated with an opioid prescription and less than a quarter of gastroenterologists wrote more than 10 neuromodulator prescriptions annually.^22^ While neuromodulators are central for the treatment of pain within the context of DGBIs, many patients still experience breakthrough pain and novel non‐opioid medications for management of acute pain attacks are still needed.^23^ In addition, the advent of brain–gut behavioral therapies has expanded our treatment armamentarium for DGBI; the robust science supporting a mechanistic link to the brain–gut axis has shown the benefit of non‐pharmacologic therapies in comprehensive pain management.^24^ Brain–gut psychotherapies can be highly customized, can be used across the spectrum of painful DGBI and augment the effect of pharmacologic treatments. The threshold to introduce brain‐gut behavioral therapies must be lowered,^25^ but the scaling of these interventions globally and to smaller communities must also be considered.

## 5 CONCLUSION

Using data from the large‐scale multinational study, the RFGES, we found that 14.8% of respondents with DGBI used prescription pain medications, about twice as high as those without a DGBI diagnosis. Despite the geographic differences in prevalence, the prevalence ratios of prescription pain medication usage in respondents with and without DGBI were notably similar, indicating that pain medication prescription patterns in DGBI are influenced by overall national prescribing behaviors. Environmental, sociodemographic, and individual factors may influence clinicians to consider personalized, multimodal approaches to address pain in patients with DGBI.

## AUTHOR CONTRIBUTIONS

YL, ADS, and LAK involved in conceptualization. YL, SAC, SIB, OSP, and ADS involved in methodology. YL contributed to writing—original draft. YL, SAC, OSP, ADS, and LAK contributed to writing—review and editing. All authors have reviewed and approved the final draft to be submitted.

## FUNDING INFORMATION

The Rome Foundation Global Epidemiology Study was funded, in part, by research grants from Ironwood, Shire, Allergan, and Takeda. The study in Israel was funded by Takeda‐Israel. The study in Romania was funded by the Romanian Society of Neurogastroenterology. None of the funders were involved in the planning, design, implementation, statistical analyses, or any other aspect of the study including the preparation of the paper or knowledge of its contents. No specific funding was received for this study.

## CONFLICT OF INTEREST

Laurie Keefer is a consultant to Pfizer and Abbvie and is a co‐founder/equity owner for Trellus Health.
